# Effect of Wholegrain Flour Particle Size in Bread on Glycaemic and Insulinaemic Response among People with Risk Factors for Type 2 Diabetes: A Randomised Crossover Trial

**DOI:** 10.3390/nu13082579

**Published:** 2021-07-27

**Authors:** Evelyn Mete, Jillian Haszard, Tracy Perry, Indrawati Oey, Jim Mann, Lisa Te Morenga

**Affiliations:** 1 Department of Human Nutrition, University of Otago, Dunedin 9054, New Zealand; evelyn.mete@otago.ac.nz (E.M.); jill.haszard@otago.ac.nz (J.H.); jim.mann@otago.ac.nz (J.M.); 2 Department of Food Science, University of Otago, Dunedin 9054, New Zealand; indrawati.oey@otago.ac.nz; 3 Riddet Institute, Palmerston North 4442, New Zealand; 4 Division of Sciences, University of Otago, Dunedin 9054, New Zealand; tracy.perry@otago.ac.nz; 5 Department of Medicine, University of Otago, Dunedin 9054, New Zealand; 6 Research Centre for Hauora and Health, Wellington Campus, Massey University, Wellington 6140, New Zealand

**Keywords:** grain particle size, wholegrain flour, stone-milled flour, stoneground flour, glycaemia, acute blood glucose, whole grain, digestion, metabolism, insulin

## Abstract

Wholegrain flour produced by roller-milling is predominantly comprised of fine particles, while stoneground flour tends to have a comparatively smaller proportion of fine particles. Differences in flour particle size distribution can affect postprandial glycaemia in people with type 2 diabetes and postprandial insulinaemia in people with and without type 2 diabetes. No prior studies have investigated the effect of wholegrain flour particle size distribution on glycaemic or insulinaemic response among people with impaired glucose tolerance or risk factors for type 2 diabetes. In a randomised crossover study, we tested the 180-min acute glycaemic and insulinaemic responses to three wholegrain breads differing in flour particle size and milling method: (1) fine roller-milled flour, (2) fine stoneground flour, and (3) coarse stoneground flour. Participants (*n* = 23) were males and females with risk factors for type 2 diabetes (age 55–75 y, BMI >28 kg/m^2^, completing less than 150 min moderate to vigorous intensity activity per week). Each test meal provided 50 g available carbohydrate, and test foods were matched for energy and macronutrients. There was no significant difference in blood glucose iAUC (incremental area under the curve) between the coarse stoneground flour bread and the fine stoneground flour bread (mean difference −20.8 (95% CI: −51.5, 10.0) mmol·min/L) and between the coarse stoneground flour bread and the fine roller-milled flour bread (mean difference −23.3 (95% CI: −57.6, 11.0) mmol·min/L). The mean difference in insulin iAUC for fine stoneground flour bread compared with the fine roller-milled flour bread was −6.9% (95% CI: −20.5%, 9.2%) and compared with the coarse stoneground flour bread was 9.9% (95% CI: −2.6%, 23.9%). There was no evidence of an effect of flour particle size on postprandial glycaemia and insulinaemia among older people with risk factors for type 2 diabetes, most of whom were normoglycaemic.

## 1. Introduction

Since carbohydrate sources like bread and grain-based dishes are staple foods in the cuisines of people worldwide, it is important to examine how the benefits traditionally associated with wholegrain consumption are influenced by modern food processing methods. Wholegrain consumption can improve acute postprandial glycaemia compared with refined grains [1] and is associated with reduced risk of developing type 2 diabetes [2,3] and other non-communicable diseases [3]. More specifically, the grain particle size of wholegrain foods can affect acute postprandial glycaemia and insulinaemia [3,4,5,6,7,8,9,10], so it is an important determinant of the health benefits of wholegrain foods. However, grain particles are not uniformly sized, and therefore, a wholegrain food or ingredient could include a wide range of wholegrain particles from very tiny to fully intact grains. Wholegrain breadmaking flours can vary substantially in terms of particle sizes depending on the way they are milled. The most common way to make flour is through roller milling, during which the whole grains pass quickly through a series of rollers and are crushed and sieved to produce a very fine flour. An alternative method grinds the grains between two millstones to produce a coarser flour, commonly referred to as stoneground flour. Few studies have actually measured the metabolic response to different wholegrain flour [3,6,8,9]. Bread made with stoneground or coarse wholegrain flour results in a lower acute postprandial glycaemic response among people with type 2 diabetes compared to bread made with roller-milled wholegrain flour [3,8], but the same effect has not been shown for people with normal glucose tolerance [6,9,11]. However, consumption of foods made from coarse compared with finely milled flours can result in reduced postprandial insulinaemia in both people with diabetes and those with normal glucose tolerance [6,8,9].

Another group for whom we have limited knowledge about the metabolic effects of grain particle size is people with prediabetes or impaired glucose tolerance (IGT). This group comprises a large (7.7%) and growing proportion of the world’s population [12,13], approximately 70% of whom will eventually progress to type 2 diabetes [14]. But through healthy lifestyle changes, people with IGT are able to return to a state of normal glucose tolerance [14] or avoid progressing to type 2 diabetes [15,16]. Providing specific advice about carbohydrate quality could be one way that dietitians and health professionals could help individuals diagnosed with prediabetes to avoid diabetes.

To address this gap in knowledge about the benefits of processed wholegrains, the aim of this study was to measure the acute metabolic response to breads made from roller-milled wheat flour and two stoneground wheat flours, each differing in their grain particle size distribution, among people who are overweight and obese, inactive, and older in age, as these factors are associated with having prediabetes, insulin resistance, and increased risk of type 2 diabetes [12,17,18,19].

## 2. Materials and Methods

This randomised, acute, controlled crossover trial was designed to test the 180-min blood glucose and insulin responses to three wholegrain breads differing in flour particle size among participants with known risk factors for type 2 diabetes. Each participant underwent a meal test on three separate mornings in randomised order to test the acute glycaemic and insulin responses to the three wholegrain wheat breads made with (1) fine roller-milled flour, (2) fine stoneground flour, and (3) coarse stoneground flour.

Using a standard deviation of the difference for iAUC (incremental area under the curve) of 152 mmol·min/L [3], the sample size required to detect a difference in blood glucose iAUC of 100 mmol·min/L between conditions with 80% power to alpha = 0.05 level was 21 participants.

Participants were randomised to the order that they would receive the test foods using a Williams square design [20], which ensures that each test food is preceded and followed by each other test food in equal numbers. The recruitment aim was 24 participants so that each of the four randomisation orders for the Williams square design would have an equal number of participants allocated to it. Each randomisation order was sealed in an opaque envelope and was randomly allocated to each participant by one of the researchers. If a participant dropped out, their randomisation order was allocated to the next new participant to maintain a balanced Williams square design.

### 2.1. Participants

Participants were males and females with known risk factors for type 2 diabetes aged 55–75 years old, BMI > 28 kg/m^2^, and not meeting New Zealand’s physical activity guidelines of >150 min moderate to vigorous intensity activity per week [12,17,18,19]. Exclusion criteria were previous diagnosis of diabetes, HbA1c measurement at or over 50 mmol/mol, current chronic infection, taking medication known to affect or control blood glucose, weight change greater than 2% in the previous 3 months, blood donation within previous 12 weeks, avoidance of or allergy to wheat or gluten, current attempt to lose or gain weight, and unwillingness or inability to comply with intervention requirements. Participants were recruited from Dunedin, New Zealand, through email, flyers, social media, newspapers, and personal connections. Ethical approval was granted by the University of Otago Human Ethics committee (H19/098), and the trial is registered at the Australia New Zealand Clinical Trials Registry (ACTRN12619001142123). All participants provided informed written consent.

### 2.2. Screening

People who expressed interest in participation and confirmed that they met the study eligibility criteria were invited to attend a screening visit held at the University of Otago Human Nutrition Clinic. Participants provided information on demographics, medical conditions, medications taken, and allergies and intolerances. Duplicate measures were taken for body weight to the nearest 0.1 kg, height to the nearest 0.1 cm using a calibrated stadiometer, and waist circumference to the nearest 0.1 cm (measuring the narrowest point between the iliac crest and lower ribs). Systolic and diastolic blood pressure was taken near the end of the screening visit after participants had been seated for 10 or more minutes and was measured by a digital automatic blood pressure monitor (Omron, HEM-907, Osaka, Japan), using the average of three consecutive tests taken in direct succession. Glycated haemoglobin (HbA1c) was measured in capillary blood from a finger prick with the Tina-quant Haemoglobin A1c Gen.2 kit, using the hemolysate application. These were analysed using a cobas c 311 (Roche Diagnostics; Basel, Switzerland).

### 2.3. Test Meals

Test meals consisted of bread providing 50 g available carbohydrate (approximately 3–4 slices) served with 250 mL water. Breads were isoenergetic and were matched for macronutrient composition, water, and sodium.

Test breads, varying only by the type of wholegrain flour used, were prepared in the food-grade laboratories at the Department of Food Science (University of Otago) by baking six loaves per batch. Ingredients are listed in Supplemental Table S1. Kneading, rising, and baking times were identical for all breads. Portions containing 50 g available carbohydrate were frozen at −20 °C. Portions were defrosted overnight in a refrigerator (4 °C) when required and were removed from the refrigerator 45 min before consumption.

All flour was defined as wholegrain in that the bran, germ, and endosperm were all present in the same relative proportions as they exist in the original, intact grain [21]. Flour was sourced from two manufacturers: roller-milled flour and coarse stoneground flour was sourced from one manufacturer, and fine stoneground flour was sourced from a second manufacturer. Each flour was produced commercially using the supplier’s own processes and practices. The milling process influenced the particle size distribution of the three different flours. Flour particle size was determined by sieve analysis [22]. The proportion of grain retained on sieves of defined sizes was measured by a mechanical sieve that held sieves that retained flour at 63, 90, 125, 180, 250, 355, 500, 710, 1000, and 1400 μm. Samples of flour (40 g) were added to the topmost sieve (1400 μm sieve), and the mechanical shaker was run for ten minutes. The grains left on each sieve were weighed to assess the grain particle size distribution (Table 1). The particle size distribution of four wholegrain stoneground flours was determined (Supplemental Table S2), and two were chosen for inclusion in the present study.

Nutrient composition of the three test breads is described in Table 2. Freshly made and cooled bread samples were vacuum sealed and sent to an independent laboratory (Assure Quality) for nutrient analysis. Carbohydrate was calculated by difference. Water content was determined by loss on drying.

### 2.4. Study Protocol

Participants arrived at the clinic (University of Otago, Dunedin, New Zealand) between 07:00 and 07:30 a.m., having fasted at least for ten hours. Participants chose which mornings to attend for testing, with the restriction that they were not able to choose to attend on consecutive days, giving a minimum washout period of at least one full day. They participated in a total of three tests on three different mornings testing the three different breads. Participants were blinded to which of the test breads they consumed at each visit. On the day prior to each test morning, participants were instructed to avoid alcohol, to choose the same evening meal prior to each test morning, and to keep consistent the duration and intensity of any physical activity undertaken. Upon arrival at each testing session, adherence to these conditions was confirmed, and if the conditions were not met, the participant was rescheduled.

Participants had a cannula (BD^®^ Insyte Autoguard shielded I.V. Catheter 20G) placed into a forearm vein by a trained health professional for sampling of blood insulin and a one-off sample to measure glycated haemoglobin. After cannulation, participants remained seated for ten or more minutes, and then blood samples were taken for baseline blood glucose and insulin measurements: two capillary blood samples were taken within 5 min via fingerprick to assess fasting blood glucose concentration, and a 2 mL venous blood sample was taken via the cannula to measure fasting insulin concentration. Immediately after baseline tests, participants were given their test meal and 250 mL water to consume within 10 min or as close to this time as they could reasonably manage. The time taken to finish eating the test meal was recorded. Blood glucose and insulin concentration were measured via a single fingerprick and a 2 mL venous blood draw, respectively, at 15, 30, 45, 60, 90, 120, 150, and 180 min from the time of meal commencement. Alcohol swabs were used to clean the finger prior to each fingerprick. Participants remained seated unless they needed to visit the bathroom in an adjacent room.

Capillary blood glucose concentrations were immediately analysed using a HemoCue Glucose 201+ analyser. Each HemoCue Glucose 201+ analyser was tested against the Glucotrol^®^-NG controls (Levels 1, 2, and 3), and outcomes were within the specified ranges.

To measure plasma insulin, each 2 mL venous sample was drawn directly into an EDTA-coated vacutainer (BD^®^ Vacutainer K3EDTA). Immediately prior to the venous blood collection, 1 mL of blood was drawn from the cannula and discarded to ensure that the sample did not include blood trapped in the cannula. After each 2-mL sample was collected, the cannula was cleaned with a 3 mL saline flush. Venous blood samples were held during the test morning for up to 3.5 h in an insulated container containing an ice pack. Blood samples were then centrifuged, and plasma samples were stored in duplicate at −80 °C for up to 4 months. Plasma insulin was measured with a Elecsys Insulin kit (Cat. No. 12017547) using a Cobas e 41 analyser (Roche Diagnostics; Basel, Switzerland). The coefficients of variation for the Elecsys Insulin Kit were 2.31% and 2.04% for the low and high controls.

### 2.5. Statistical Analysis

Statistical analyses were conducted using Stata/IC 16.1 (StataCorp, College Station, TX, USA).

Incremental area under the blood glucose curve (iAUC) was calculated over 180 min using the trapezoidal method, ignoring the area below baseline. The same method was used to determine insulin iAUC except that it was calculated over 150 min because venous blood samples at 180 min were not available for several participants due to blood clotting and blocking the cannula.

Mean differences in blood glucose and insulin iAUC between test foods were estimated using mixed effects regression models with blood glucose or insulin iAUC as the dependent variable, test food as the independent variable, and participant as a random effect. Insulin iAUC values were log-transformed for analysis to improve homoskedasticity of the model residuals. Model estimates were back-transformed and presented as percentage differences in the dependent variable between test meals. For both blood glucose and insulin iAUC, robust variance structure was used. Variables for randomisation order and time taken to eat the test food were also included in the models. Adjustment for these variables was decided *a priori*, since time taken to eat is known to affect postprandial glycaemia [25], and order group is a design element [26]. Mean differences, 95% CI, and *p*-values were calculated. *p* < 0.05 was considered statistically significant with no adjustment for multiple tests.

We also conducted an exploratory sensitivity analysis to determine whether having features of glucose intolerance moderated the effect of flour particle size on postprandial blood glucose and insulin iAUC because of the high level of heterogeneity in glucose tolerance status amongst the participants. Participants categorised as having features of impaired glucose tolerance had either or both of fasting blood glucose ≥ 6.1 mmol/L or HbA1c ≥ 40 mmol/mol. One participant with normal glycated haemoglobin (34 mmol/mol) was categorised as having features of glucose intolerance because they were clearly hyperinsulinaemic (with all insulin iAUCs above the 95th percentile of the group data), and their fasting blood glucose was considered to be impaired (5.6 mmol/L) according to the American Diabetes Association [27]. The mixed-effects regression models for blood glucose and insulin iAUC were repeated with stratification by whether or not participants had features of glucose intolerance.

## 3. Results

**Figure 1:**
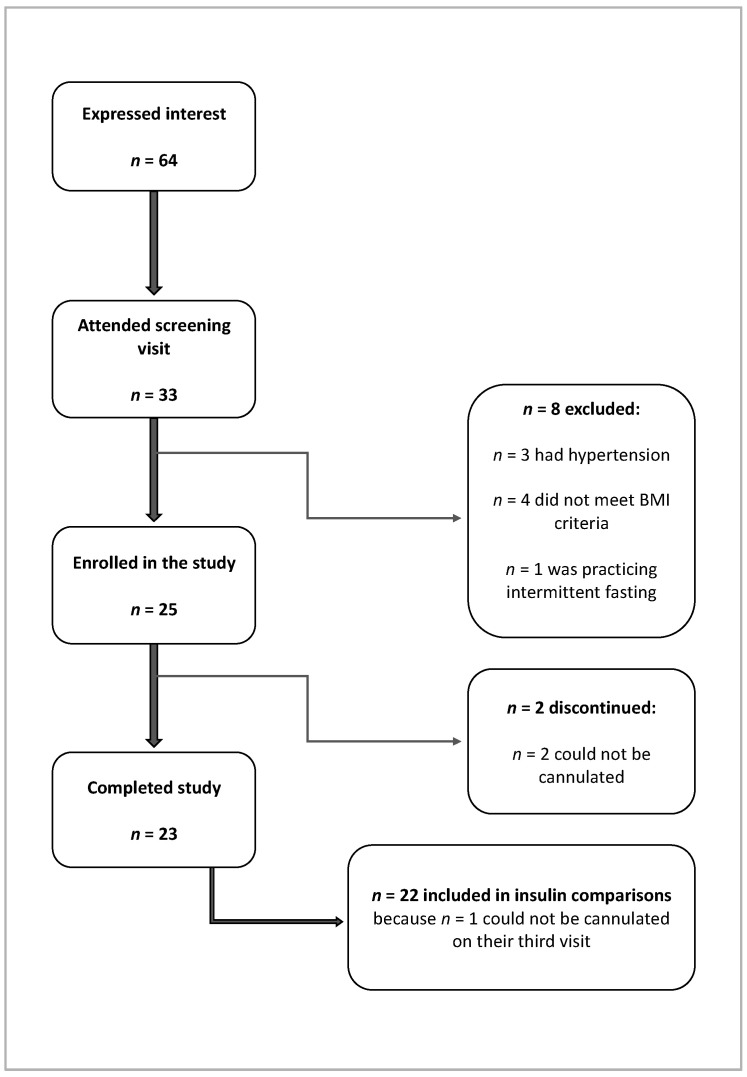
Participant flowchart.

Thirty-three participants attended a screening visit, but eight of these were excluded from participation (Figure 1). Hypertension was not an initial exclusion criterion, but for ethical reasons, a decision was made to advise that participants with both systolic and diastolic blood pressure higher than 160 and 100 mmHg, respectively, should prioritise treating their hypertension without delay. Two participants could not complete the study because they could not be cannulated for blood sampling. Twenty-three participants completed the study.

Eleven participants (48%) took medications for cardiovascular disease. Participants self-reported that they engaged in a median of 20 min of moderate to vigorous intensity exercise per week (25th, 75th percentiles: 0, 100 min). Fasting blood glucose ranged from 4.2 to 7.4 mmol/L. Nineteen participants had obesity (BMI ≥ 30 kg/m^2^), and four had a BMI > 28 and <30 kg/m^2^. Further details of participant characteristics are described in Table 3.

When considering the group as a whole, there was no evidence of differences in blood glucose iAUC or insulin iAUC (Table 4) in response to any of the three breads. The blood glucose iAUC for coarse stoneground flour bread was not significantly different from the fine stoneground flour bread (mean difference −20.8 (−51.5, 10.0) mmol·min/L) and from the fine roller-milled flour bread (mean difference −23.3 (−57.6, 11.0) mmol·min/L). The insulin iAUC for fine stoneground flour bread was not significantly different from for the fine roller-milled flour bread (% difference (95% CI) = −6.9% (−20.5%, 9.2%)), and the insulin iAUC for the coarse stoneground flour bread was not significantly different from the fine roller-milled flour bread (% difference (95% CI) = 9.9% (−2.6%, 23.9%).

Graphs showing the mean glycaemic and insulinaemic responses to test meals are available in Supplemental Figures S1 and S2. However, they do not depict the within-person comparisons; therefore, they are illustrative only.

In an exploratory sensitivity analysis, those with features of glucose intolerance (*n* = 8) had a marginally larger mean difference in glycaemic response between bread made from coarse stoneground flour and fine roller-milled flour (mean difference (95% CI) = 37.5 (−34.2, 109.2) mmol·min/L) and between bread made from coarse stoneground flour and fine stoneground flour (mean difference (95% CI) = 39.5 (−22.4, 101.4) mmol·min/L) (Table 5) compared with the mean differences shown in the primary analysis (Table 4). Amongst those with features of glucose intolerance, the differences in insulin iAUC between breads were amplified compared with the findings from the primary analysis (Table 5).

## 4. Discussion

Among people with known risk factors for type 2 diabetes, there were no significant differences in glycaemic or insulinaemic response to breads made with coarse stoneground flour, fine stoneground flour, and fine roller-milled flour, although the glycaemic response to the bread made from coarse flour was marginally lower than the two breads made from fine flour. When results were stratified by degree of glucose intolerance status, the differences in glycaemic response between the coarse stoneground flour bread and the two breads made from fine flour were amplified among people with features of glucose intolerance (*n* = 8). These differences were not statistically significant, and nor were the subgroup sample sizes sufficient to detect significance. Since the exploratory sensitivity analysis relies on a small and poorly defined sample, these findings are speculative and cannot inform a definitive conclusion. In contrast, insulin iAUC outcomes did not follow the trends observed for blood glucose iAUC for either the primary analysis or the sensitivity analysis. Consumption of both the roller-milled flour bread and the coarse stoneground flour bread resulted in a higher insulinaemic response compared to fine stoneground flour bread in the primary analysis (*n* = 23). These differences were larger among those with features of glucose intolerance (*n* = 8).

The proportion of larger grain particles in wholegrain flours may be more important than the proportion of fine and ultrafine particles as a determinant of postprandial glycaemic response. In contrast with our findings, the two prior studies, which found a statistically significant effect of flour particles size on glycaemia in people with type 2 diabetes [3,8], used coarse stoneground flours that had a substantially greater proportion (40%) of particles larger than 1000 µm [8] and a smaller proportion (9%) of fine (<150 µm) particles [3] compared with the coarse stoneground flour used in our study (approximately 20% < 150 µm and 17% > 710 µm). Many fine (<150–180 μm) and most ultra-fine (<90 μm) wheat flour particles will have a larger proportion of damaged starch and more damaged cell walls compared to coarser particles (approx. 330–700 μm), resulting in faster starch hydrolysis [23]. It is possible that our relatively high proportion (20%) of fine and ultra-fine particles in the coarse stoneground flour counteracted the effect of the larger particles on glycaemic response. Our findings, in conjunction with other published literature [3,8], suggest that describing a wholegrain food by its mean or median grain particle size may provide insufficient information for identifying foods that minimise postprandial glycaemic responses. Instead, for optimal health benefits, manufacturers of wholegrain flour and similar ingredients could consider minimising the proportion of fine particles while maximising the proportion of large particles. It may be particularly important to minimise the proportion of particles below 150–180 μm in diameter.

Our findings did not provide evidence that varying the proportion of ultra-fine particles (<90 μm) in wholegrain flour affects postprandial glycaemia or insulinaemia. The proportion of ultra-fine particles differed between all three flour types used to make our test breads, whereas the proportion of fine flour (<180 μm) was similar between the fine stoneground flour and the fine roller-milled flour (Table 1). Our finding was congruent with a previous study that also did not detect a difference in postprandial glycaemia and insulinaemia among normoglycaemic participants following consumption of breads made from fine flour versus ultra-fine flour [11].

Our unexpected findings for postprandial insulinaemia did not align with findings from prior studies [6,8,9]. In our study, there is no clear explanation for why the insulin iAUC was slightly lower after consuming bread made from fine stoneground flour than for the bread made from coarse stoneground flour. Given that a previous study showed that the glycaemic and insulinaemic indices of mixed cereal-based meals were not correlated [28], it is possible that our findings were simply a chance finding and represent no trend at all. Alternately, insulinaemic response may be a poorer marker of metabolic response among insulin insensitive participants.

Stoneground flour was chosen for the coarser flour options because it typically is comprised of a larger proportion of coarser particles than fine particles [3,29]. However, our particle size analysis of four commercially available wholegrain stoneground flours demonstrates that stoneground flours vary considerably in their grain particle-size distribution (Supplemental Table S2). In contrast with our findings in a previous study, stoneground flour had a larger percentage of small particles (<85 µm) compared with roller-milled flour [30].

A limitation of this study is that we did not obtain information on the cultivars of our wheat and the temperature that the stone mills attained during milling since we used commercially available flours. Heat generated during stone milling can vary from 35 °C to 90 °C [31,32,33], and heat damage is associated with an increased proportion of damaged starch for some wheat varieties. The effect of heat on the extent of starch damage has the potential to affect starch digestibility and, therefore, the acute glycaemic response. It is possible that our stoneground flours may have greater starch damage than the roller-milled flour, which may have also increase postprandial glycaemia and, therefore, counteract the potential effect of grain particle size. However, it is also worth noting that substantial starch damage may occur without high heat during milling [34]. The proportion of damaged starch is inversely related to particle size, and ultra-fine particles have the largest proportion of damaged starch compared to larger particles [23,32]. Other factors, including grain moisture content, the amount of space between millstones, and the grain-feed rate in stone milling, all influence starch damage, resulting in variation in the proportion of damaged starch ranging from 6–72% [35]. For future research, it may be worth involving observations of the milling process and obtaining measures of damaged starch.

It is possible that factors beyond that of particle size alone may have contributed to the lack of significant differences in glycaemic and insulinaemic responses among the breads. For practical reasons, our breads were frozen and subsequently thawed overnight in a refrigerator, so formation of resistant starch would be expected [36]. It is not known whether the effect of resistant starch on glycaemia and insulinaemia interacts with the effect of grain particle size. However, our findings represent a realistic situation where free-living individuals may store bread in the freezer or refrigerator. Another factor that may have affected our findings is that the three flours were sourced from two separate manufacturers, and the wheat varieties were not known.

There is lack of evidence for the importance of grain particle size for people with prediabetes. Our study partially contributes towards building this evidence base, but it would have been stronger had we been able to recruit people with prediabetes. Given that people are frequently unaware of having prediabetes, we considered that the cost and time involved in screening for participants who met the diagnostic of prediabetes or impaired glucose tolerance would have been prohibitive. The post hoc analysis suggests that a future study should confirm whether people with prediabetes respond differently to the effects of grain particle size compared to normoglycaemic people. It should also be noted that this sensitivity analysis was underpowered and was only carried out for exploratory purposes. Therefore, the findings may be useful to hypothesise future research directions but not to draw conclusions from.

## 5. Conclusions

Many consumers consider stone-milled flour to be more desirable, as it is perceived as a natural product [37]. Our research showed that stoneground flours produced by different mills varied considerably in their particle size distribution, and therefore, not every wholegrain stoneground flour may have the potential to reduce glycaemia in those with type 2 diabetes or impaired glucose tolerance. If wholegrain stoneground flour were to be recommended as an alternative to roller-milled flour for management of glycaemia, it would be essential to also regulate its particle size distribution.

From our research, it does not appear that those with normoglycaemia have additional benefits relating to postprandial glycaemia or insulinaemia from choosing wholegrain bread products made with coarse stoneground flour rather than finer roller-milled flour. However, the findings from our exploratory sensitivity analysis contribute to the basis for a future study to confirm whether people with prediabetes will benefit from coarser flour, in line with what has been shown in other studies amongst people with type 2 diabetes [3,8]. Given that a quarter of New Zealanders have prediabetes [12], it is important to determine whether using larger flour particle size to improve bread quality could reduce postprandial glycaemia and thereby contribute to prevention of progression to type 2 diabetes.

## Figures and Tables

**Table 1:** Flour particle size distribution of roller-milled flour, fine stoneground flour, and coarse stoneground flour.

Size Categories	Grain Size	Roller-Milled Flour	Fine Stoneground Flour	Coarse Stoneground Flour
Very very coarse	% ≥1.4 μm	0.8	0.2	0.2
Very coarse	% 710–1399 μm	5.6	1.9	16.6
Coarse	% 356–709 μm	9.2	9.5	35.7
Medium	% 181–355 μm ^a^	2.1	11.1	22.5
Fine	% 91–180 μm	31.6	47.8	11.3
Ultra-fine	% ≤90 μm ^b^	47.7	25.2	12.3
	% Lost ^c^	2.9	4.4	1.4

^a^ Larger than the average size of a wheat endosperm cell (100–150 μm) [23]. ^b^ Smaller than the average size of a wheat endosperm cell (100–150 μm) [23]. ^c^ The sieve stack was not airtight, and it is likely that the lost portion was comprised of fine particles.

**Table 2:** Nutrient analysis of breads made from roller-milled flour, fine stoneground flour, and coarse stoneground flour.

	Roller-Milled Flour Bread (g/100 g)	Fine Stoneground Flour Bread (g/100 g)	Coarse Stoneground Flour Bread (g/100 g)
Ash	1.9	1.9	2.1
Carbohydrate by difference	35.0	35.8	34.0
Energy (kJ)	938	942	933
Dietary fibre	7.2	7.4	8.6
Moisture	42.4	41.7	41.5
Protein ^a^	10.8	10.7	11.2
Starch	30.8	28.5	29.3
Fat	2.7	2.5	2.6

^a^ A protein conversion factor of 5.83 was used to convert nitrogen into protein content, as is appropriate for wholegrain wheat [24].

**Table 3:** Participant characteristics.

Participant Characteristics		Mean (SD)
Age (years)		63.6 (5.5)
Sex (% female)		74%
HbA1c (mmol/mol) ^a^		36.4 ^b^
BMI (kg/m^2^)		35.9 (6.7)
Systolic blood pressure (mmHg)		133 (22)
Diastolic blood pressure (mmHg)		80 (10)
Waist circumference (cm)	Males	109.4 (4.2) ^c^
	Females	106.6 (11.5) ^d^
Fasting blood glucose (mmol/L) ^e^		5.2 (0.8)

^a^ HbA1c was available for 22 of the 23 participants. Due to researcher error, a sample for measuring HbA1c was not collected from one participant. ^b^ Since there were 8 participants with features of glucose intolerance and 14 with normal glucose tolerance, HbA1c was not normally distributed. The median was 36.5 mmol/mol (25th and 75th percentiles: 31–40). ^c^ Missing waist circumference for one of the six male participants. ^d^ Missing waist circumference for one of the seventeen female participants. ^e^ Mean of fasting blood glucose across the three test days.

**Table 4:** Blood glucose 180-min iAUC (*n* = 23) and insulin 150-min iAUC (*n* = 22) for breads prepared with differing flour-milling method and particle size.

	**Mean (SD)**	**Mean Difference (95% CI) ^a^ from Fine Roller-Milled Flour Bread**	***p*-Value**	**Mean Difference (95% CI) ^a^ from Fine Stoneground Flour Bread**	***p*-Value**
Blood glucose iAUC (mmol × min/L)					
Fine roller-milled flour	282 (108)	Reference			
Fine stoneground flour	279 (101)	−3 (−38, 33)	0.889	Reference	
Coarse stoneground flour	259 (76)	−23 (−58, 11)	0.184	−21 (−52, 10)	0.185
	**Geometric Mean (95% CI)**	**Percent ^c^ Difference (95% CI) from Fine Roller-Milled Flour Bread**	***p*-Value**	**Percent ^c^ Difference (95% CI) from Fine Stoneground Flour Bread**	***p*-Value**
Insulin iAUC ^b^ (µU × min/mL)					
Fine roller-milled flour	8416 (6273, 11,289)	Reference			
Fine stoneground flour	7811 (5873, 10,390)	−6.8% (−20.5%, 9.2%)	0.380	Reference	
Coarse stoneground flour	8671 (6762, 11,119)	2.3% (−8.3%, 14.2%)	0.681	9.9% (−2.6%, 23.9%)	0.125

^a^ Adjusted for randomisation order and time to eat. ^b^ Insulin iAUC (incremental area under the curve) was calculated using the 0–150 min measures due to missing data. ^c^ Mean percent differences are presented because insulin iAUC values were log transformed for analysis, then back-transformed.

**Table 5:** Sensitivity analysis for blood glucose 180-min and insulin 150-min iAUC for breads prepared with differing flour-milling method and particle size in which only participants with features of glucose intolerance (*n* = 8) are included.

	**Mean (SD)**	**Mean Difference (95% CI) ^a^ from Fine Roller-Milled Flour Bread**	***p*-Value**	**Mean Difference (95% CI) ^a^ from Fine Stoneground Flour Bread**	***p*-Value**
Blood glucose iAUC (mmol·min/L)					
Fine roller-milled flour	310 (151)	Reference			
Fine stoneground flour	314 (119)	−2 (−70, 66)	0.954	Reference	
Coarse stoneground flour	272 (71)	38 (−34, 109)	0.305	40 (−22, 101)	0.211
	**Geometric Mean (95% CI)**	**Percent ^c^ Difference (95% CI) from Fine Roller-Milled Flour Bread**	***p*-Value**	**Percent ^c^ difference (95% CI) from Fine Stoneground Flour Bread**	***p*-Value**
Insulin iAUC ^b^ (µU·min/mL)					
Fine roller-milled flour	11,444 (6055; 21,627)	Reference			
Fine stoneground flour	9989 (5362; 18,609)	−13.5% (−23.1%, −2.8%)	0.015	Reference	
Coarse stoneground flour	11,322 (6317; 20,293)	−1.1% (−13.2%, 12.8%)	0.874	14.3% (−5.2%, 38.0%)	0.161

^a^ Adjusted for randomisation order and time to eat. ^b^ Insulin iAUC was calculated using the 0–150 min measures due to missing data. ^c^ Mean percent differences are presented because insulin iAUC values were log transformed for analysis, then back-transformed.

## Data Availability

Data supporting reported results are available upon request from the corresponding author.

## Supplementary Materials

The following are available online at https://www.mdpi.com/article/10.3390/nu13082579/s1, Table S1: Ingredients of breads made from fine roller-milled flour, fine stoneground flour, and coarse stoneground flour. Table S2: Flour particle size distribution of one wholegrain roller-milled wheat flour and four wholegrain wheat stoneground flours. Figure S1: Mean postprandial capillary blood glucose responses to wholegrain breads of different milling method and grain particle size among participants with known risk factors for type 2 diabetes (*n* = 23). Figure S2: Mean postprandial insulin responses to wholegrain breads of different milling method and grain particle size among participants with known risk factors for type 2 diabetes (*n* = 23).

## Clinical Trial Registration

Australia New Zealand Clinical Trials Registry (ACTRN12619001142123).
