# Supplementary material for: Effect of Wholegrain Flour Particle Size in Bread on Glycaemic and Insulinaemic Response among People with Risk Factors for Type 2 Diabetes: A Randomised Crossover Trial
Source: Nutrients. 2021 Jul 27;13(8):2579. doi: 10.3390/nu13082579 (PMC8398996; doi:10.3390/nu13082579)
Supplement: Supplementary file 1 [file nutrients-13-02579-s001.zip › Supplemental Figure 2.pdf]

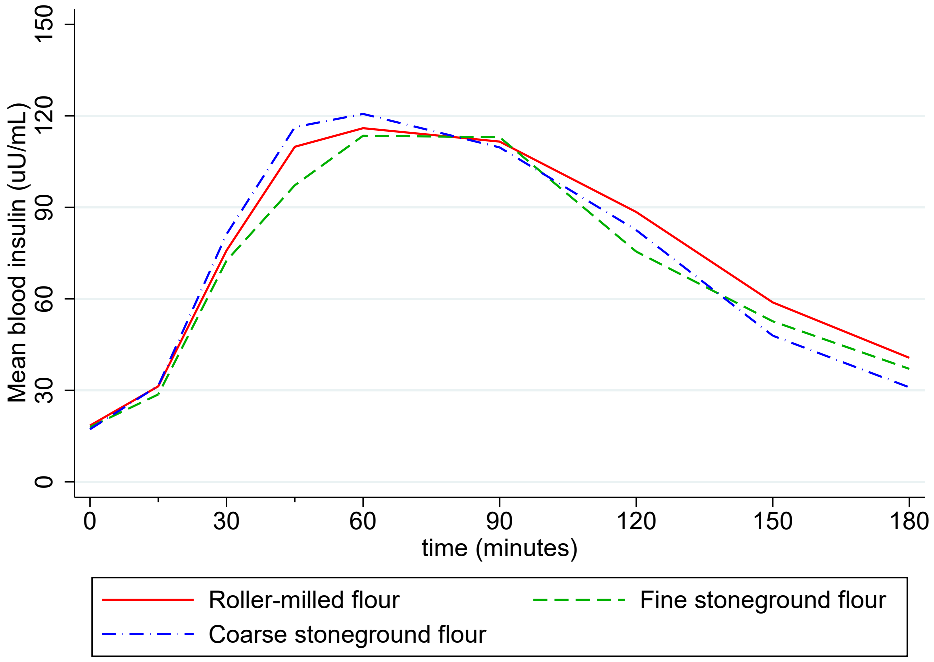

Supplementary Figure S2: Mean postprandial insulin responses to wholegrain breads of different milling method and grain particle size among participants with known risk factors for type 2 diabetes (n=23).

This figure is intended to illustrate postprandial insulinaemic response patterns; error bars are not included to avoid inappropriate inference given this is a crossover trial.
