# Supplementary material for: Effect of Wholegrain Flour Particle Size in Bread on Glycaemic and Insulinaemic Response among People with Risk Factors for Type 2 Diabetes: A Randomised Crossover Trial
Source: Nutrients. 2021 Jul 27;13(8):2579. doi: 10.3390/nu13082579 (PMC8398996; doi:10.3390/nu13082579)
Supplement: Supplementary file 1 [file nutrients-13-02579-s001.zip › Supplemental Table 1.pdf]

Supplementary Table S1: Ingredients of breads made from fine roller-milled flour, fine stoneground flour, and coarse stoneground flour

Ingredient	% of total	Quantity per batch[a]
**Wholegrain wheat flour[b]**	55%	3223.8
**Water**	39%	2321.4
**Salt**	1%	48.6
**Improver & Softener**	<1%	32.4
**Gluten**	3%	193.2
**Calcium acetate**	<1%	19.2
**Vegetable oil**	<1%	32.4
**Yeast**	<1%	18.0

[a] Each batch produced six loaves.

[b] Fine roller-milled flour, fine stoneground flour, and coarse stoneground flours were used for each of the three breads.
