# Supplementary material for: Effect of Wholegrain Flour Particle Size in Bread on Glycaemic and Insulinaemic Response among People with Risk Factors for Type 2 Diabetes: A Randomised Crossover Trial
Source: Nutrients. 2021 Jul 27;13(8):2579. doi: 10.3390/nu13082579 (PMC8398996; doi:10.3390/nu13082579)
Supplement: Supplementary file 1 [file nutrients-13-02579-s001.zip › Supplemental Table 2.pdf]

Supplementary Table S2: Flour particle size distribution of one wholegrain roller-milled wheat flour and four wholegrain wheat stoneground flours

Sieve size	Roller-milled flour[b]	Stoneground Flour A[b]	Stoneground Flour B[b]	Stoneground Flour C[c]	Stoneground Flour D[c]
**% >1.4 mm**	0.8	0.2	0.2	0	0.9
**% 1 – 1.4 mm**	2	3.6	1.9[a]	0.8	9.4
**% 710 μm – 999 μm**	3.5	13		6.8	17.7
**% 500 – 709 μm**	5.5	19	5.2	15.8	15.6
**% 355 – 499 μm**	3.7	16.7	4.3	17.4	10.1
**% 250 – 354 μm**	1	13.8	3.7	15.8	7.8
**% 180 – 249 μm**	1.1	8.7	7.4	9.3	5.2
**% 125 – 179 μm**	9.6	6.8	19.8	8.2	5
**% 90 – 124 μm**	22	4.5	28	6	4.7
**% 63 – 89 μm**	21.8	4.7	11.4	5.6	6
**% <63 μm**	25.9	7.6	13.8	11.9	15.4
**% Lost[d]**	2.9	1.4	4.4	2.5	1.9
**% over 710 μm**	6.45	16.7	2.2	31.7	28.1
**% below 180 μm**	79.3	23.7	73	23.4	31.1

[a] The 1 mm sieve was not included in the sieve stack for this flour; so the category is 710 – 1399 μm.

[b] Used to make breads for glycaemic and insulinaemic testing in the present study

[c] Not included in glycaemic and insulinaemic testing in the present study

[d] The sieve stack was not airtight and it is likely that the 'lost' portion was comprised of fine particles.
